# Task-Evoked Pupillary Dynamics Are Altered in Post-COVID Syndrome

**DOI:** 10.3390/medsci14020269

**Published:** 2026-05-21

**Authors:** Alexander Smit, Philipp Fleischmann, Thomas S. Knauer, Christian Y. Mardin, Georg Michelson, Julia Zott, Moritz Güttes, Helena Sarmiento, Miriam Ilgner, Marie Jakobi, Jürgen Rech, Bettina Hohberger

**Affiliations:** 1 Department of Ophthalmology, University Hospital, Friedrich Alexander University Erlangen–Nuremberg, 91054 Erlangen, Germany; 2 Talkingeyes & More, Erlangen, 91052 Erlangen, Germany; 3 Department of Internal Medicine 3-Rheumatology and Immunology, Deutsches Zentrum Immuntherapie and Center for Rare Diseases Erlangen, Universitätsklinikum Erlangen, Friedrich-Alexander-Universität Erlangen-Nürnberg, 91054 Erlangen, Germany

**Keywords:** Post-COVID syndrome, Long COVID, pupillometry, cognitive load, Index of Pupillary Activity (IPA), Low/High Index of Pupillary Activity (LHIPA), autonomic nervous system, virtual reality-based assessment

## Abstract

**Background/Objectives:** Post-COVID syndrome (PCS) is frequently associated with persistent cognitive complaints such as fatigue and impaired concentration, yet objective markers related to cognitive dysfunction are lacking. Pupillary oscillation metrics have emerged as non-invasive indicators of task-related cognitive load and autonomic regulation. This study investigated the Index of Pupillary Activity (IPA) and the Low/High Index of Pupillary Activity (LHIPA) in a large cohort of patients with PCS compared with healthy controls. **Methods:** In this cross-sectional study, 526 participants (397 PCS patients, 129 controls) performed a standardized virtual reality-based stereoscopic task at three disparity levels: 275 arcsec (high difficulty), 550 arcsec (medium difficulty), and 1100 arcsec (low difficulty), using a head-mounted display with integrated eye tracking. Continuous pupillometry data were recorded, and IPA and LHIPA were calculated. Linear mixed-effects models with random intercepts for participants were applied, adjusting for age, sex, and task difficulty. **Results:** Both IPA and LHIPA were significantly lower in PCS patients than in controls at all three task difficulty levels in post hoc model-based contrasts. In adjusted mixed-effects models, PCS was also associated with lower overall IPA (β=−0.111, 95% CI −0.160 to −0.062, p<0.001) and lower overall LHIPA (β=−0.164, 95% CI −0.253 to −0.074, p<0.001). Lower task difficulty was associated with higher values of both metrics: for IPA, β=0.164 at 550 arcsec and β=0.287 at 1100 arcsec (both p<0.001); for LHIPA, β=0.161 at 550 arcsec and β=0.254 at 1100 arcsec (both p<0.001), relative to 275 arcsec. Thus, both indices showed an inverse association with task difficulty. Age was negatively associated with both metrics, whereas male sex was positively associated with both. No significant interaction between cohort and task difficulty was observed. **Conclusions:** PCS was associated with reduced IPA and LHIPA during a standardized stereoscopic task. These findings indicate altered task-related pupillary dynamics in PCS and may reflect altered cognitive-load processing and autonomic regulation. LHIPA, and with caution also IPA, may contribute to the objective assessment of task-related pupillary alterations in PCS.

## 1. Introduction

PCS is a clinical symptom complex after infection with Severe Acute Respiratory Syndrome Coronavirus 2 (SARS-CoV-2). It is characterized by novel or persistent symptoms that last at least three months after acute Coronavirus disease 2019 (COVID-19), in the absence of an alternative diagnosis [1]. Fatigue and concentration difficulties are among the most frequently reported symptoms [2]. However, objective physiological markers of cognitive dysfunction in PCS remain limited, complicating diagnosis, stratification, and monitoring in both clinical care and research. PCS remains a major challenge for both clinicians and patients because objective diagnostic markers and causal therapeutic options are still lacking [3,4]. The prevalence data for PCS vary or are even missing for distinct regions [5,6]. PCS is increasingly understood as a heterogeneous syndrome rather than a single clinical entity, comprising overlapping subgroups such as autonomic dysregulation, immune-mediated pathways, organ-specific sequelae (e.g., lung fibrosis, myocarditis, and irritable bowel syndrome), and vascular dysfunction [7,8,9,10,11,12,13,14]. As sympathetic and parasympathetic pathways contribute to pupil function, it can be assumed that an autonomic dysfunction might be mirrored by altered pupil dynamics. The control of pupil size and reaction is regulated by two muscles: the M. sphincter, regulated by the parasympathetic nervous system (cholinergic neuron), and the M. dilator pupillae, innervated by the sympathetic nervous system (not cholinergic). The opposing constriction of the respective muscles causes the pupil to constrict (miosis) or dilate (mydriasis).A distinct regulation of the pupil is necessary to enable a rapid adaptation of the visual system to different light conditions [15,16].

Pupillometry is an ophthalmic method based on a non-invasive and quick measurement of pupillary response. Previously, pupillometry has been successfully used in patients with familial dysautonomia and autonomic neuropathies [17]. Recent data suggest that patients with PCS also show an altered pupillary response: a reduction of duration of pupil constriction (n=25 patients with PCS) [18], an altered pupillary response after different lighting conditions (n=44 patients with PCS) [19], or a reduction in pupil dilation and constriction (n=65 patients with PCS, requiring hospitalization during acute COVID-19) [20].

Those studies of the pupillary light reflex primarily assess reflexive autonomic and brainstem-mediated responses to luminance changes, whereas task-evoked pupil fluctuations reflect cognitive effort, arousal regulation, and mental workload during active task performance [21,22]. In PCS, previous pupillometric studies have mainly described abnormalities in light-evoked or basic pupillary responses, including altered contraction dynamics and reduced dilation/constriction responses [18,19,20]. Task-evoked oscillatory pupil metric seems to be an interesting approach as an objective marker for cognitive dysfunction in PCS.

To assess task-evoked pupil dynamics related to cognitive load, the IPA was proposed as a frequency-based measure of rapid pupil diameter oscillations during task performance [21]. The LHIPA further relates low- and high-frequency components of the pupil signal. Because luminance-related changes and baseline drift typically occur on slower timescales than task-evoked microdilations, these indices aim to emphasize cognitively driven pupil dynamics while reducing contamination by ambient light variation [23]. Recent data suggest that IPA may reflect cognitive impairment in PCS during a virtual 3D task [24]. In addition, subsequent machine learning analyses identified IPA and LHIPA among the most informative features to distinguish patients with PCS from healthy controls [25]. Therefore, the present study examined IPA and LHIPA in a large cohort of patients with PCS and healthy controls during a standardized virtual-reality stereoscopic task with graded difficulty. By combining two frequency-based pupillary metrics with a controlled task paradigm and mixed-effects modeling, this study aimed to examine whether task-evoked pupil oscillations differ between patients with PCS and healthy controls and whether these measures may serve as objective indicators of altered cognitive load processing in PCS.

## 2. Materials and Methods

### 2.1. Study Cohort

A total of 526 participants were recruited at the Department of Ophthalmology, University Hospital Erlangen, Germany: 397 patients with PCS (41.6% male, 58.4% female) and 129 healthy controls (49.6% male, 50.4% female). Mean age was 40.6 ± 13.2 years and did not differ markedly between the cohorts (controls: 36.8 ± 15.5 years; PCS: 41.9 ± 12.2 years). Mean time for PCS duration was 677.1 ± 347.2 days. SARS-CoV-2 infection was confirmed by a positive polymerase chain reaction. Demographic data are presented in Table 1. In addition, functional impairment was assessed using the Bell score. The Bell score is a patient-reported outcome measurement ranging from 0 to 100 points, with higher scores indicating better functional capacity and lower symptom burden. A score of 100 reflects no symptoms at rest or during physical exertion and normal activity, whereas a score of 0 indicates severe symptoms at rest, bedridden status, and inability to perform even minor self-care activities. Exclusion criteria were pre-existing ocular disorders, systemic disorders with ocular involvement, and a best-corrected visual acuity lower than 0.8. Healthy participants had either no COVID-19 in their history or were convalescent after COVID-19 without any PCS symptoms.

### 2.2. VR-OTS

The Virtual-Reality-Oculomotor-Test-System (VR-OTS; Talkingeyes & More, Erlangen, Germany) was used to quantify visual task performance and pupillary dynamics under stereoscopic viewing conditions. The system was implemented in a head-mounted display (HTC Vive Pro Eye, HTC Corporation, Taoyuan, Taiwan) with two 3.5-inch OLED displays (1440×1600 pixels per eye), a refresh rate of 90 Hz, a nominal field of view of $110^{\circ }$, and adjustable interpupillary distance (IPD; 60–73 mm). Binocular eye tracking was provided by the integrated Tobii system (Tobii AB, Danderyd, Sweden), specified by the manufacturer at 120 Hz.

**Figure 1:**
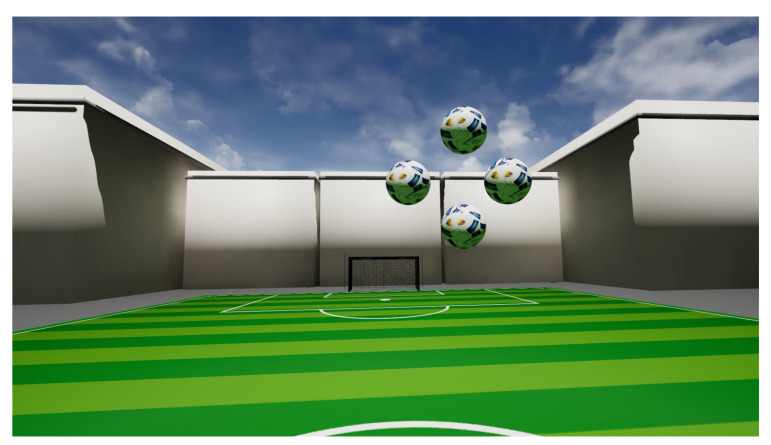
Screenshot of the virtual reality-based stereoscopic task implemented in the VR-OTS. Four balls are displayed in a rhomboid arrangement, with one ball rendered closer than the others by a defined binocular disparity. In this example, the left ball is perceived as closest to the viewer at a disparity level of 550 arcsec. The stereoscopic depth effect is perceived during binocular presentation in the VR headset and is not apparent in the 2D screenshot. Participants identified the closest ball using the corresponding arrow key.

The virtual scene consisted of a stadium-like environment, in which four balls were presented at a virtual viewing distance of 200 cm in a rhomboid arrangement (see Figure 1). Each ball had a virtual diameter of 25 cm, and the distance from each ball to the center of the arrangement was 25 cm. The stimulus could appear at nine predefined positions within the visual field: one central position and eight peripheral positions (down, lower right, right, upper right, up, upper left, left, and lower left). Peripheral locations were generated using spherical coordinates with a fixed inclination angle of $10^{\circ }$.

In each stereoscopic stimulus, one of the four balls was rendered closer to the participant than the other three balls. Task difficulty was manipulated by binocular disparity, using three disparity levels: 275, 550, and 1100 arcsec. The 275-arcsec condition represented the smallest disparity difference that could be implemented with the display geometry of the headset. Smaller disparity values correspond to higher task difficulty because the depth difference between the balls becomes harder to resolve. Participants were instructed to identify the ball perceived as closest and to indicate their response with the corresponding arrow key on a wired keyboard as quickly and accurately as possible. Before testing, participants were introduced to the headset and task. A 5-point eye-tracker calibration was then performed using the HTC SRanipal runtime (version 1.3.6.8); this procedure was also used to optimize headset positioning and adjust the IPD setting. Participants who usually wore spectacles performed the test without spectacles in order to avoid a reduction in eye-tracking accuracy.

Each participant completed three runs. At the beginning of each run, all four balls were presented at the same depth (idle phase). After the participant initiated the run by pressing an arrow key, 81 stereoscopic stimuli were presented in randomized order. These stimuli comprised all combinations of 3 disparity levels, 9 stimulus positions, and 3 repetitions. The first run served as familiarization, the second as training, and data from the third run were used for statistical analysis. The duration of the analyzed third run was defined as the total time required to complete the 81-stimulus stereoscopic task.

For each stimulus, the system recorded response time (time from stimulus onset to key press), response correctness, disparity level, and stimulus position. Simultaneously, binocular pupil diameter, gaze direction, gaze origin, and eye openness were recorded continuously during task performance using the SRanipal plugin for Unreal Engine. In the original technical implementation, eye-tracking data were sampled synchronously with the rendering loop of the virtual environment; the headset hardware itself provides binocular eye tracking at 120 Hz.

Pupillometry preprocessing followed the previously described VR-OTS preprocessing pipeline [24,26]. Blink-contaminated and invalid samples were removed prior to feature extraction. Blinks were identified using the eye-openness signal; samples with eye openness <0.1 were classified as blinks. Pupil samples outside the physiological range of 1.5–9.0 mm were rejected. Additional artifact rejection included removal of samples with implausibly large sample-to-sample changes, samples strongly deviating from a fitted trend line, and temporally isolated short clusters. Thereafter, the mean pupil diameter across both eyes was computed, and missing samples were interpolated using a Piecewise Cubic Hermite Interpolating Polynomial approach.

After preprocessing, the mean binocular pupil diameter signal was used as the input signal for the pupillary oscillation metrics. The IPA and LHIPA were computed within the VR-OTS software (version 1.0.0) environment and used as device-derived pupillometric indices. The algorithmic basis of these indices follows the wavelet-based approaches described by Duchowski et al. for IPA [21] and LHIPA [23]. Therefore, the following description summarizes the computational principles underlying these device-derived measures.

IPA quantifies short-term oscillatory activity in the pupil diameter signal. In brief, the preprocessed pupil diameter time series is decomposed using a discrete wavelet transform, allowing rapid local changes in pupil diameter to be identified in the wavelet detail coefficients. Local maxima of the wavelet coefficient signal are then used to detect abrupt small-scale pupil diameter changes, and thresholding is applied to suppress minor fluctuations likely attributable to noise. The remaining suprathreshold events are counted and normalized by the duration of the analyzed signal segment. Accordingly, IPA is not expressed in millimeters of pupil diameter but represents a time-normalized frequency-like index of detected pupillary oscillation events. Higher IPA values therefore indicate a greater number of rapid pupil diameter fluctuations per unit time.

LHIPA is computed from the same preprocessed pupil diameter signal but extends the IPA concept by considering the relationship between lower- and higher-frequency components of pupil oscillation. After wavelet decomposition, coefficients from a low-frequency band and a high-frequency band are related to obtain a low-/high-frequency ratio of pupil activity. Modulus maxima are then detected in this ratio signal, thresholded, counted, and normalized by the signal duration. LHIPA is therefore also a time-normalized oscillatory index rather than a direct measure of pupil diameter. Because LHIPA is based on the ratio between low- and high-frequency components, lower LHIPA values have been interpreted as reflecting relatively stronger high-frequency pupillary oscillations and thus greater task-related pupillary unrest [23].

In the present study, IPA and LHIPA were computed for the stimulus epochs of the analyzed run and averaged within each disparity level. The three disparity conditions therefore provided task-specific summary values of pupil oscillatory activity for each participant. The resulting IPA and LHIPA values were used as relative indices of task-evoked pupillary dynamics across cohort and disparity-defined task difficulty levels.

Brightness within the virtual scene was kept constant by using the same programmed VR environment for all participants. In the original system description, monocular lighting cues were minimized by avoiding cast shadows and by using a distant single light source in the virtual environment. To reduce potential effects of ambient light entering from outside the virtual environment, care was taken to ensure a close-fitting and standardized positioning of the VR headset before each measurement.

### 2.3. Statistical Analysis

All statistical analyses and data visualization were performed in Python (version 3.13.7) using scipy (version 1.16.3), statsmodels (version 0.14.5), and seaborn (version 0.13.2). Descriptive statistics were computed at the participant level. Continuous variables are reported as mean ± standard deviation (SD) and median with interquartile range (IQR), whereas categorical variables are reported as counts and percentages.

The distribution of the pupillary outcome measures, IPA and LHIPA, were assessed separately for each cohort (PCS and control) and each disparity level (275, 550, and 1100 arcsec). Normality was evaluated using the Shapiro–Wilk test. In addition, boxplots and histograms with kernel density overlays were inspected visually to assess distributional shape and the presence of marked skewness or outliers. The density overlays were computed using a Gaussian kernel with bandwidth selected according to Scott’s rule and no additional bandwidth adjustment.

To account for the repeated-measures structure of the data, linear mixed-effects models (LMMs) with random intercepts for participants were fitted separately for IPA and LHIPA. For this purpose, each participant contributed up to three repeated observations corresponding to the three disparity levels. The dependent variable was the respective pupillary measure (IPA or LHIPA). Fixed effects included cohort (PCS vs. control), disparity level (275, 550, and 1100 arcsec), sex (female vs. male), and age. An interaction term between cohort and disparity level was included to test whether the effect of task difficulty differed between cohorts. Control cohort, 275 arcsec disparity, and female sex served as the reference categories. Age was centered around the sample mean prior to model fitting. The resulting model is defined in Equation (1).

(1) $$\begin{array}{cc} Y_{ij}= & \beta_{0}+\beta_{1}\;{\mathrm{Cohort}}_{i}+\beta_{2}\;{\mathrm{Disparity}}_{ij}+\beta_{3}\;\left({\mathrm{Cohort}}_{i}\times {\mathrm{Disparity}}_{ij}\right)\\ \; & +\beta_{4}\;{\mathrm{Sex}}_{i}+\beta_{5}\;{\mathrm{Age}}_{c,i}+b_{0i}+\varepsilon_{ij}, \end{array}$$

where $Y_{ij}$ denotes the observed outcome value (IPA or LHIPA) for participant *i* at disparity level *j*, $\beta_{0}$ is the fixed intercept, $\beta_{1}$ represents the fixed effect of cohort, $\beta_{2}$ the fixed effect of disparity level, $\beta_{3}$ the cohort-by-disparity interaction, $\beta_{4}$ the fixed effect of sex, and $\beta_{5}$ the fixed effect of centered age. The term $b_{0i}$ denotes the participant-specific random intercept, accounting for within-subject dependence across repeated measurements, and $\varepsilon_{ij}$ is the residual error term. The random intercepts and residuals were assumed to follow normal distributions.

For the mixed-effects analyses, one summary value per participant and disparity level was entered into the model. Thus, each participant contributed three observations (275, 550, and 1100 arcsec), resulting in 526×3=1578 observations in total.

Model diagnostics were assessed by visual inspection of residual-versus-fitted plots, Q–Q plots, histograms of residuals, and residual distributions across cohorts and disparity levels. This inspection indicated mild leptokurtic tendencies, with residuals in the periphery exceeding the values expected under a normal distribution, which is not uncommon in clinical datasets. However, linear mixed-effects models are generally considered robust to minor deviations from normality of this kind, particularly in sufficiently large samples. Given the sample size of 526 participants, these deviations were not considered substantial enough to invalidate the model-based inference.

Models were estimated using restricted maximum likelihood (REML). Fixed effects are reported as regression coefficients (β) with corresponding 95% confidence intervals (CI) and two-sided *p*-values. Statistical significance was defined as p<0.05. In addition to the main mixed-model analyses, simple cohort effects were estimated within each disparity level using model-based contrasts. Because these comparisons were performed separately within each outcome, *p*-values were adjusted using the Holm method. Model-based predicted values were derived from the fitted mixed-effects models for graphical presentation.

## 3. Results

### 3.1. Distributional Characteristics of Pupillary Indices

**Figure 2:**
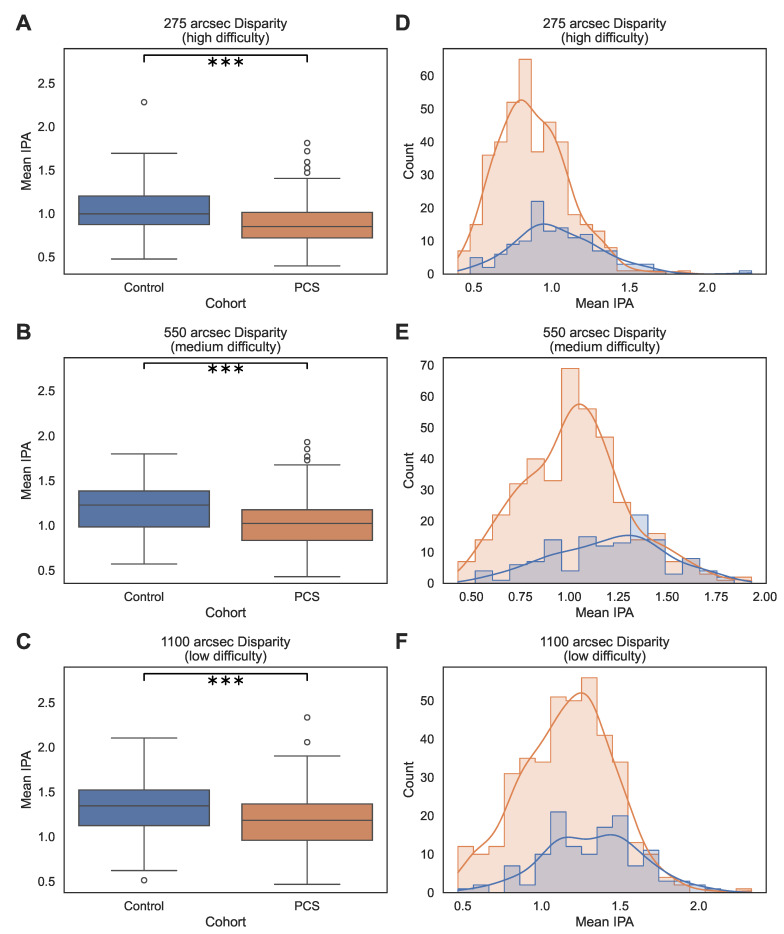
Distribution of IPA across cohorts and disparity-defined task difficulty levels. Panels (**A**–**C**) show boxplots of mean IPA values for the control and PCS cohorts at 275 arcsec (high difficulty), 550 arcsec (medium difficulty), and 1100 arcsec (low difficulty), respectively. Boxes represent the IQR with median lines; whiskers extend to 1.5× the IQR, and circles indicate individual data points beyond the whiskers. Brackets with asterisks indicate the significance of the cohort comparison at the respective disparity level based on Holm-adjusted *p*-values (*** p<0.001). Panels (**D**–**F**) show the corresponding histograms with kernel density estimates for the same disparity levels. Blue represents the control cohort and orange represents the PCS cohort; bars indicate the frequency distribution, and solid lines indicate the kernel density estimates.

**Figure 3:**
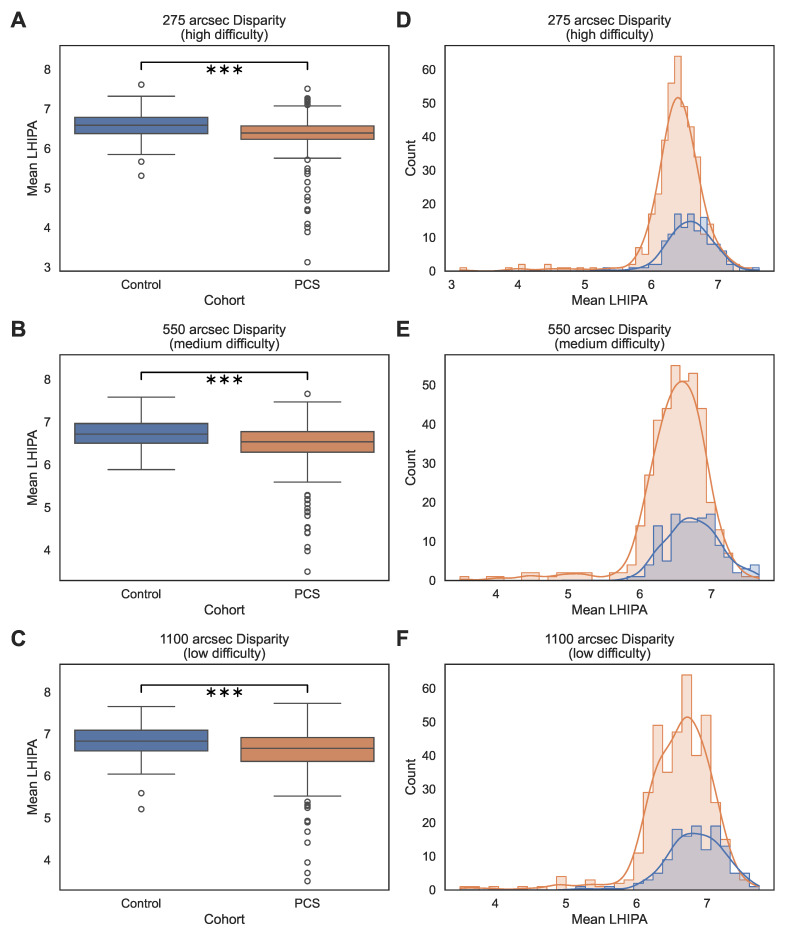
Distribution of LHIPA across cohorts and disparity-defined task difficulty levels. Panels (**A**–**C**) show boxplots of mean IPA values for the control and PCS cohorts at 275 arcsec (high difficulty), 550 arcsec (medium difficulty), and 1100 arcsec (low difficulty), respectively. Boxes represent the IQR with median lines; whiskers extend to 1.5× the IQR, and circles indicate individual data points beyond the whiskers. Brackets with asterisks indicate the significance of the cohort comparison at the respective disparity level based on Holm-adjusted *p*-values (*** p<0.001). Panels (**D**–**F**) show the corresponding histograms with kernel density estimates for the same disparity levels. Blue represents the control cohort and orange represents the PCS cohort; bars indicate the frequency distribution, and solid lines indicate the kernel density estimates.

Across all participants, the analyzed third run was completed in a median time of 99.07 s, with an IQR of 78.12 s to 131.06 s and a mean duration of 114.31 s ± 56.28 s. Box plots and kernel density estimates (Figure 2 and Figure 3) illustrate the distributions of both the IPA and LHIPA values, stratified by cohort and disparity, respectively. Across all disparity levels, both metrics showed unimodal distributions with approximately symmetric shapes, particularly at higher disparities. Formal Shapiro–Wilk testing indicated deviations from strict normality for several subgroup disparity combinations, especially in the PCS cohort. Kernel density plots demonstrated approximate normal distributions, and the large sample size per condition supported the use of LMMs for inferential analysis [27].

### 3.2. Linear Mixed-Effects Model for IPA

Linear mixed-effects modeling revealed lower IPA values in patients with PCS compared with healthy controls (see Table 2).

**Figure 4:**
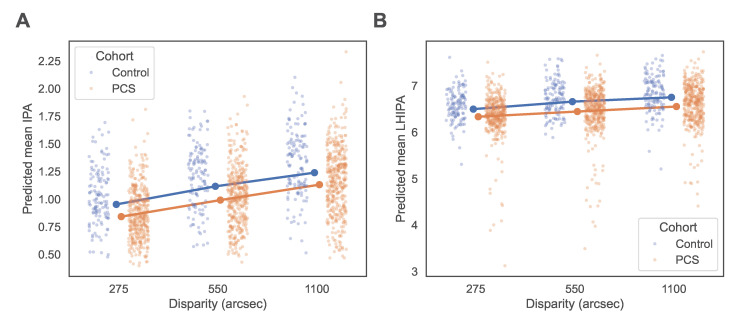
Age- and sex-adjusted predicted means of pupillary indices across disparity levels and cohorts. Predicted mean IPA (**left**) and predicted mean LHIPA (**right**) were derived from linear mixed-effects models with random intercepts for participants. Values are shown for the control and PCS cohorts across the three disparity levels (275, 550, and 1100 arcsec), corresponding to high, medium, and low task difficulty, respectively. Points represent model-adjusted means at centered age ($Age_{c}=0$) and female sex; lines illustrate disparity-related trends within each cohort. Post hoc model-based contrasts showed significantly lower values in the PCS cohort than in controls at all disparity levels for both IPA and LHIPA (all Holm-adjusted p<0.001).

The model included a random intercept for participant and fixed effects for cohort, disparity, sex, centered age, and the cohort-disparity interaction. IPA values were significantly lower in the PCS cohort compared with controls (β=−0.111, 95% CI −0.160 to −0.062, p<0.001). Increasing disparity was associated with higher IPA values, with significant effects for both 550 arcsec (β=0.164, p<0.001) and 1100 arcsec (β=0.287, p<0.001) relative to 275 arcsec. Thus, IPA showed an inverse association with task difficulty, with lower values in the highest-difficulty condition. Male sex was associated with higher IPA values (β=0.109, p<0.001), while increasing age showed a significant negative association with IPA (β=−0.008 per year, p<0.001). The interaction between cohort and disparity was not significant in the joint Wald test for the interaction term ($\chi^{2}\left(2\right)=0.89$, p=0.640), indicating similar disparity-dependent changes in IPA across cohorts. Age- and sex-adjusted predicted means derived from the model are shown in Figure 4 and Table A1. Post hoc model-based contrasts showed that the PCS cohort had significantly lower IPA values than controls at all three disparity levels. At 275 arcsec, the adjusted mean difference was β=−0.111 (95% CI [−0.160,−0.062], Holm-adjusted p<0.001); at 550 arcsec, β=−0.125 (95% CI [−0.174,−0.075], Holm-adjusted p<0.001); and at 1100 arcsec, β=−0.109 (95% CI [−0.158,−0.060], Holm-adjusted p<0.001; see Table A2).

In the PCS cohort, Bell score was not significantly correlated with mean IPA (Spearman’s ρ=0.081, p=0.114).

### 3.3. Linear Mixed-Effects Model for LHIPA

Analysis of the LHIPA using linear mixed-effects modeling revealed significantly lower values in the PCS cohort compared with controls (see Table 3). As with the IPA model, a random intercept for participant was included. LHIPA values were significantly lower in the PCS cohort compared with controls (β=−0.164, 95% CI −0.253 to −0.074, p<0.001). Higher disparities were associated with increased LHIPA values at both 550 arcsec (β=0.161, p<0.001) and 1100 arcsec (β=0.254, p<0.001), respectively. Male sex was positively associated with LHIPA (β=0.106, p=0.003), while age showed a significant negative association (β=−0.007 per year, p<0.001). No significant cohort-by-disparity interaction was observed in the joint Wald test ($\chi^{2}\left(2\right)=2.15$, p=0.342). Adjusted predicted means for LHIPA by cohort, disparity, and sex are displayed in Figure 4 and Table A1.

Post hoc model-based contrasts likewise showed significantly lower LHIPA values in the PCS cohort than in controls at all three disparity levels. At 275 arcsec the adjusted mean difference was β=−0.164 (95% CI [−0.253,−0.074], Holm-adjusted p<0.001); at 550 arcsec, β=−0.213 (95% CI [−0.303,−0.124], Holm-adjusted p<0.001); and at 1100 arcsec, β=−0.199 (95% CI [−0.289,−0.110], Holm-adjusted p<0.001; see Table A2). In the PCS cohort, the Bell score was not significantly correlated with mean LHIPA (Spearman’s ρ=0.088, p=0.087).

## 4. Discussion

PCS, long-COVID, or post-acute sequelae are terminologies defining ongoing and life-restricting symptoms after acute COVID-19. There is a high demand for visualization and quantification of the wide range of diverse patient-reported symptoms. Recent data suggest that IPA and/or LHIPA might offer an objective indicator for quantification of the impaired cognitive load in PCS [24,25]. After adjustment for age, sex, and task difficulty, LHIPA showed lower values in PCS than in controls in the mixed-effects models, arguing for an increased cognitive load; post hoc model-based contrasts confirmed significantly lower values at each disparity level. Yet, IPA was observed to fail during this 3D-task environment.

Pupillometry is an ophthalmic method based on a non-invasive measurement of pupil size for assessing cognitive and autonomic function. In addition to regulating retinal illuminance, pupil size is linked to arousal and mental effort. Research shows that changes in the pupil area scale with mental effort and difficulty [28,29]. Correlations between transient pupil dilations and mental load or difficulty were observed [21]. These rapid movements are independent of the slower pupillary light reflex and are considered an expression of spontaneous mental effort. Altered pupillary light reflexes and lowered dilation speed were observed during acute COVID-19 [30]. Further PCS patients showed a lower pupil diameter and a higher pupillary unrest index, both linked to a reduced central nervous activation [31,32]. Traditional measurements based on mean or peak diameter are highly susceptible to baseline drift because of brightness changes limiting applicability [33,34]. Thus, in order to emphasize pupillary dynamics, IPA was established as a signal-processing-based value accentuating high-frequency changes in pupil diameter [23]. Not referencing overall pupil area, IPA quantifies the rate of rapid pupillary movement, also known as pupillary unrest or micro-dilations, within a certain time frame. IPA indirectly quantifies change in task-evoked mental load through high-frequency pupillary fluctuations while minimizing baseline shift caused by environmental brightness as well as exhaustion-related baseline shifts [21].

LHIPA was introduced by Duchowski et al., offering a marker for cognitive load in settings where IPA failed [23]. It was specifically proposed as a more robust low/high-frequency ratio-based metric, decreasing with increasing cognitive load. IPA is a sensitive marker for a fixed-gaze task, which is based on mental arithmetic. Yet, creating an n-back task, a test including the working memory, IPA is not able to show the cognitive load, contrary to LHIPA. Sensitive results of LHIPA were shown during an n-back test while the eye could move. The results of the present study confirm these data. LHIPA was observed to show sensitively the cognitive load in patients with PCS during a 3D task, while IPA failed to do so.

Pupil function is regulated by the autonomic nervous system. Parasympathetic postganglionic fibres originating from neurons in the ciliary and pterygopalatine ganglia, project to the sphincter pupillae muscle, controlling pupil constriction (cholinergic neurons). Sympathetic postganglionic fibers (non-cholinergic neurons), originating from the superior cervical ganglion, project to the dilator pupillae muscle, controlling pupil dilation. In addition to the brainstem and autonomic mechanisms, the pupil diameter is affected by both visual and non-visual cortical regions. Small pupil diameter changes during higher order cortical functions (e.g., problem solving) were observed, even when the stimuli do not contain a change in the viewing distance or retinal illuminance but vary by color, spatial frequency, and apparent motion. These pupillary movements are closely linked to neuromodulatory mainly noradrenergic and cholinergic influences. Noradrenaline (norepinephrine) mainly causes quick, short pupil dilations via the locus coeruleus. Acetylcholine causes longer-lasting pupil changes [16,35,36]. Reduced pupil dilation is consistent with parasympathetic dysfunction and brainstem function in charge of pupil control, being present during acute infections and after clinical recovery indicating persisting effects [37]. PCS pathophysiological mechanism of an impaired microvascular tissue blood supply is linked to an autonomic and brainstem dysfunction [38]. Reduced microvascular supply might induce metabolic dysregulation in the autonomic nervous system, which may account for the lower IPA and LHIPA values reported in the submitted work. Altered pupil dynamics measured during mental load in patients with PCS suggest a pathological altered behavior of the cognitive function [20,22].

It is assumed that cognitive PCS symptoms were related to autonomic nervous system dysfunctions and altered central arousal regulation, especially in the brainstem [39]. Initial evidence was obtained from autopsies of patients who died during acute COVID-19; next to microglial and macrophage activation, especially in the white matter of brainstem and cerebellar regions [40], SARS-CoV-2 RNA was observed in neuronal regions, reaching from the olfactory system to the brainstem [41]. Of interest, patients with PCS and cognitive impairments showed hypometabolic areas in the brainstem and pons in Fluorodeoxyglucose PET, independently of the severity of acute COVID-19 [42].

Common PCS symptoms are sustained mental fatigue, attentional deficits, impaired working memory, and slowed cognitive processing months after acute SARS-CoV-2 infection [18,30]. These symptoms are often not traceable in conventional neuroimaging or clinical neurological examinations; thus, there is a need for suitable sensitive biomarkers. As LHIPA was observed to be a sensitive marker in patients with PCS, the present data confirm previous findings [25]. Increasing task difficulty was associated with lower values of both metrics in both cohorts. Accordingly, the highest-difficulty condition (275 arcsec) showed the lowest LHIPA values, whereas the lowest-difficulty condition (1100 arcsec) showed the highest values. Age showed a negative association, as described in the literature [43,44]. Male sex was associated with higher values with an overall inconsistent study situation regarding sex-related differences in pupillometry [43,45,46,47,48].

LHIPA is associated with the locus coeruleus-norepinephrine (LC-NE) system, a key regulator of attention, arousal, and effort, with dysregulation being connected to fatigue, reduced mental efficiency, and instability [49,50]. The LC-NE system influences LHIPA via variations in noradrenergic signaling. LC neuron activation releases norepinephrine (NE), binding to adrenergic receptors, regulating neuronal excitability and synaptic gain, influencing mental effort and arousal state [51]. NE acts via G protein-coupled adrenergic receptors (including α and β subtypes), influencing intracellular cascades as cAMP-dependent signaling and modulation of ion channels [52]. Increased LHIPA during tasks could be a sign of compensatory upregulation of LC-NE activity, while increased mental effort is necessary to perform the task with reduced capability. Lowered LHIPA values may indicate hypoarousal or exhaustion prominent in patients with fatigue [32].

The present study is not without limitations. Potential confounders might interfere with LHIPA data (e.g., anticholinergic medication). The study design was conducted for patients aged older than 18 years. As VR-OTS is a non-invasive, convenient, and straightforward procedure, this method could be particularly beneficial for children and adolescents. Thus, subsequent studies should expand this finding in a cohort of patients younger than 18 years. In addition, the results are indicative of a single-center study with a European patient population and are therefore not representative of the global population. As this is a cross-sectional study, further longitudinal or therapy-associated studies are recommended to validate the findings for a wider population and assess their potential usefulness in patient assessment or monitoring [53].

## 5. Conclusions

The present data show that IPA and LHIPA values were significantly lower in PCS patients than in controls across all task difficulty levels during a standardized task. These findings indicate altered task-evoked pupillary dynamics in PCS, while the LHIPA pattern is consistent with previous methodological literature. The behavior of IPA should be interpreted cautiously, as prior work suggests that this metric may be more susceptible to experimental and signal-related influences.

## Figures and Tables

**Table 1:** Cohort characteristics. Continuous variables are reported as mean ± SD and median [IQR]; categorical variables as n (%).

Variable	Level	Overall	Control	PCS
N		526	129	397
Age (years)	Mean ± SD	40.6 ± 13.2	36.8 ± 15.5	41.9 ± 12.2
	Median [IQR]	40.0 [29.0, 51.8]	30.0 [24.0, 51.0]	41.0 [32.0, 52.0]
Sex	Female, n (%)	297 (56.5%)	65 (50.4%)	232 (58.4%)
	Male, n (%)	229 (43.5%)	64 (49.6%)	165 (41.6%)
Bell score	Mean ± SD	54.5 ± 28.8	98.3 ± 5.5	41.7 ± 18.3
	Median [IQR]	40.0 [30.0, 80.0]	100.0 [100.0, 100.0]	40.0 [30.0, 50.0]

**Table 2:** Linear mixed-effects model (LMM) results for the IPA with a random intercept for participant (PatID). Estimates are restricted maximum likelihood. Reference levels: cohort = control, difficulty = 275 arcsec, sex = female. Age_*c*_ denotes mean-centered age.

Fixed Effects	Estimate	SE	*z*	*p*	95% CI
Intercept	0.954	0.024	40.185	<0.001	[0.907, 1.000]
Cohort (PCS)	−0.111	0.025	−4.411	<0.001	[−0.160, −0.062]
Difficulty (550 arcsec)	0.164	0.016	10.289	<0.001	[0.132, 0.195]
Difficulty (1100 arcsec)	0.287	0.016	18.062	<0.001	[0.256, 0.318]
Sex (male)	0.109	0.019	5.583	<0.001	[0.071, 0.147]
Cohort (PCS) × Difficulty (550)	−0.014	0.018	−0.759	0.448	[−0.050, 0.022]
Cohort (PCS) × Difficulty (1100)	0.002	0.018	0.108	0.914	[−0.034, 0.038]
Age_*c*_	−0.008	0.001	−10.868	<0.001	[−0.009, −0.007]
**Random effects**					
PatID intercept variance	0.043				
**Model fit**					
Observations (*N*)	1578				
Participants (groups)	526				
Log-likelihood	404.79				
Residual variance (scale)	0.0163				

**Table 3:** LMM results for LHIPA with a random intercept for participant (PatID). Estimates are restricted maximum likelihood. Reference levels: cohort = control, difficulty = 275 arcsec, sex = female. Age_*c*_ denotes mean-centered age.

Fixed Effects	Estimate	SE	*z*	*p*	95% CI
Intercept	6.500	0.043	150.485	<0.001	[6.416, 6.585]
Cohort (PCS)	−0.164	0.046	−3.580	<0.001	[−0.253, −0.074]
Difficulty (550 arcsec)	0.161	0.030	5.312	<0.001	[0.102, 0.221]
Difficulty (1100 arcsec)	0.254	0.030	8.381	<0.001	[0.195, 0.314]
Sex (male)	0.106	0.035	3.023	0.003	[0.037, 0.175]
Cohort (PCS) × Difficulty (550)	−0.050	0.035	−1.422	0.155	[−0.118, 0.019]
Cohort (PCS) × Difficulty (1100)	−0.036	0.035	−1.017	0.309	[−0.104, 0.033]
Age_*c*_	−0.007	0.001	−5.157	<0.001	[−0.009, −0.004]
**Random effects**					
Patients’ intercept variance	0.139				
**Model fit**					
Observations (*N*)	1578				
Participants (groups)	526				
Log-likelihood	−580.60				
Residual variance (scale)	0.0594				

## Data Availability

The dataset analyzed during the current study is not publicly available due to general data privacy regulations but is available from the corresponding authors upon reasonable request.

## Abbreviations

The following abbreviations are used in this manuscript:

IPA	Index of Pupillary Activity
LHIPA	Low/High Index of Pupillary Activity
VR-OTS	Virtual Reality Oculomotor Test System
PCS	Post-COVID syndrome
LC-NE	Locus coeruleus-norepinephrine
IPD	Interpupillary distance
IQR	Interquartile range
SD	Standard deviation
LMM	Linear mixed-effects model
CI	Confidence interval
NE	Norepinephrine
REML	Restricted maximum likelihood
SARS-CoV-2	Severe Acute Respiratory Syndrome Coronavirus 2

## Appendix A

**Table A1:** Predicted means for IPA and LHIPA by cohort, disparity (arcsec), and sex at mean-centered age (${\mathrm{Age}}_{c}=0$).

Outcome	Cohort	Disparity (arcsec)	Sex	Predicted Mean
**IPA**	control	275	female	0.953680
	control	275	male	1.062407
	control	550	female	1.117340
	control	550	male	1.226067
	control	1100	female	1.240978
	control	1100	male	1.349705
	PCS	275	female	0.843004
	PCS	275	male	0.951731
	PCS	550	female	0.992763
	PCS	550	male	1.101491
	PCS	1100	female	1.132281
	PCS	1100	male	1.241008
**LHIPA**	control	275	female	6.500285
	control	275	male	6.606490
	control	550	female	6.661545
	control	550	male	6.767751
	control	1100	female	6.754725
	control	1100	male	6.860931
	PCS	275	female	6.336557
	PCS	275	male	6.442763
	PCS	550	female	6.448129
	PCS	550	male	6.554335
	PCS	1100	female	6.555450
	PCS	1100	male	6.661655

**Table A2:** Post hoc model-based simple cohort effects (PCS vs. control) within each disparity-defined task difficulty level for IPA and LHIPA. Contrasts were derived from the fitted linear mixed-effects models at 275, 550, and 1100 arcsec and are reported as adjusted regression coefficients (β) with standard errors (SE), z values, 95% confidence intervals (CI), unadjusted *p* values, and Holm-adjusted *p* values.

Outcome	Diff.	Estimate	SE	z	*p*	95% CI	$p_{\mathrm{Holm}}$
IPA	275	−0.111	0.025	−4.411	<0.001	[−0.160, −0.062]	<0.001
IPA	550	−0.125	0.025	−4.965	<0.001	[−0.174, −0.075]	<0.001
IPA	1100	−0.109	0.025	−4.332	<0.001	[−0.158, −0.060]	<0.001
LHIPA	275	−0.164	0.046	−3.580	<0.001	[−0.253, −0.074]	<0.001
LHIPA	550	−0.213	0.046	−4.666	<0.001	[−0.303, −0.124]	<0.001
LHIPA	1100	−0.199	0.046	−4.357	<0.001	[−0.289, −0.110]	<0.001
